# Factors associated with faster axial elongation after orthokeratology treatment

**DOI:** 10.1186/s12886-022-02294-1

**Published:** 2022-02-08

**Authors:** Ya Qi, Lizhou Liu, Yu Li, Fengju Zhang

**Affiliations:** 1 Beijing Tongren Eye Center, Beijing Tongren Hospital, Capital Medical University, Beijing Ophthalmology and Visual Science Key Lab, #1 Dong Jiao Min Xiang, Dong Cheng District, Beijing, 100730 China; 2 Department of Ophthalmology, Beijing Children’s Hospital, Capital Medical University, National Center for Children’s Health, Beijing, China

**Keywords:** myopia children, orthokeratology, axial elongation, axial length decrease

## Abstract

**Background:**

To study the baseline factors that related to faster axial elongation after orthokeratology (OK) treatment and the characteristics of cases with axial length decrease in a group of myopia children.

**Methods:**

This is a retrospective study. The records of 73 children who had wear OK lens for at least one year were reviewed. Only the data of right eyes were included. Baseline data included: age, gender, parental myopia, refractive error, corneal power, central corneal thickness, axial length and anterior chamber depth. Corneal power, central corneal thickness, anterior chamber depth and axial length after one-year of OK lens wear were also collected. The related factors affecting axial length change were analyzed. A comparison was made on the cases of axial length increase and axial length decrease.

**Results:**

Of the 73 eyes, axial length increased by 0.18 ± 0.17 mm (*P* < 0.001) after one year of OK lens wear. Correlation analysis showed that one-year axial length change was negatively correlated with age and positively correlated with the parental myopia and baseline myopia. Stepwise multiple linear regression analysis showed that the factors associated with faster axial elongation were lower baseline myopic spherical equivalent (*P* = 0.018), higher parental myopia degree (*P* = 0.026), and younger age at the onset of lens wear. (*P* = 0.039). Nine eyes showed negative axial growth (−0.06 ± 0.04 mm), and had older initial age of lens wear, higher baseline myopic spherical equivalent, and lager baseline corneal power**,** when **c**ompared with cases of axial length increase**.**

**Conclusions:**

Myopia children with lower baseline myopic spherical equivalent, younger initial age and higher parental myopia had faster axial elongation after orthokeratology treatment. More aggressive treatment should be considered. In children with slow axial elongation, OK lens wear may lead to negative axial growth. Whether there are reasons other than central corneal thinning and choroidal thickening needs further study.

## Background

The prevalence of myopia has been increasing globally with high myopia become one of the major blinding diseases [1]. Holden et al. [2] estimated that half of the world’s population would suffer from myopia by 2050, reaching 4.758 billion, and the population with high myopia would rise to 938 million. Refractive surgery can help most myopic eyes to take off their spectacles safely and effectively. However, it cannot change the nature of myopia, nor the risk of ocular diseases associated with high myopia [3, 4]. Myopia develops with age in children and adolescents, and we should intervene it according to its characteristics of progression [5] at this age to reduce the incidence of high myopia in adulthood. Studies have shown that orthokeratology (OK) is an effective treatment to inhibit axial elongation and delay myopia progression [6–16]. However, the treatment outcome varies among individuals. The axial elongation can be fast or slow down. Investigating the rules of OK lens treatment will help us to understand the treatment characteristics of orthokeratology and find out the groups that need more active intervention. Axial length decrease after OK lens wear was not a rare phenomenon in previous studies. Quite a few studies have addressed it. However, there were no targeted researches on it. We investigated the cases of negative axial growth after OK lens wear for one year, and discussed the possible causes other than thinning of central cornea and thickening of choroid.

## Methods

### Subjects

The medical records of myopia children who came to Contact Lens Clinic for vision correction and were fitted with OK lenses successfully at Beijing Tongren Eye Center of Beijing Tongren Hospital from January 2016 to June 2018 were reviewed. The subjects that met the inclusion criteria below (Table 1) were sorted out. This research followed the Tenets of the Declaration of Helsinki and was approved by the institutional review board. Data was collected from the hospital records and no patient involvement was required.

**Table 1:** Inclusion criteria

1. No prior history of contact lens use. 2. No other methods to reduce myopia progression were used (e.g., atropine eye drops of low concentration). 3. No ocular or systemic conditions other than ametropia and no eye surgery had been done. 4. Unaided visual acuity of logMAR0.1 or better achieved within 1 month of OK lens wear. 5. 8–10 h of lens wear every night. 6. No obvious adverse reactions were observed. 7. Maintain regular follow-up appointments. 8. OK lens had been worn for at least one year at the time of review.	

### Procedure of clinical assessment and review

Orthokeratology lenses used by selected myopia children were four-zone reverse geometry lenses (DreamLite Night Lenses; Procornea Nederland B.V., Gelderland, Netherland), which were manufactured using Boston XO material with a nominal Dk of 140 × 10^−11^(cm^2^/s)(mlO_2_/ml*mmHg). The nominal central thickness of the lenses was 0.22 ~ 0.27 mm and the diameter was 9.80 ~ 11.6 mm. The subjects were fitted with the lenses according to the manufacturer’s fitting guidelines.

All patients underwent a standard anterior eye and refractive status assessment prior to fitting OK. This assessment included visual acuity, slit-lamp examinations, intraocular pressure (CT-80A, Topcon Corp., Tokyo, Japan), corneal power (KR-810, Topcon Corp., Tokyo, Japan), central corneal thickness and corneal endothelial cell density (SP-2000P, Topcon Corp., Tokyo, Japan), axial length and anterior chamber depth (IOL Master 500, Corl Zeiss Meditec, Jena, Germany), topographic evaluation (TMS-4, Tomey Corp., Nagoya, Japan).

Refractive error was measured thirty minutes after administration of the last of the four drops of 0.5% tropicamide (Santen Pharmaceutical Co. Ltd. China), separated five minutes apart in each eye, using auto-kerato-refractometer (KR-810, Topcon Corp., Tokyo, Japan). Axial length measurements were performed according to the manufacturer’s instructions. On each occasion, data from the first five successful measurements were collected, and their average was used as a representative value.

Follow up visits were scheduled for one day, one week, one month, three months, and every three months after OK lens wear.

### Data collection

Data were retrospectively collected from the clinical records of the patients that meet with the inclusion criteria. Only the right eye data were used for statistical analysis. The data collected included: age at the initial OK lens fitting, gender, parental myopia, baseline spherical equivalent (SE) refractive error, corneal power, central corneal thickness (CCT), anterior chamber depth (ACD), and axial length (AL). In addition, corneal power, CCT, ACD, and AL after one-year OK lens wear were collected too.

### Statistical analysis

Measurements used for data analysis were obtained only from the right eye of each participant. IBM SPSS statistics version 21.0 (IBM Co., Armonk, NY, USA) was used for all the statistical analyses. The distribution of each parameter was assessed using the Kolmogorov–Smirnov test. For normally distributed variables, statistical comparisons between groups were made using the paired t-test, one-way analysis of variance (ANOVA), and associations were analyzed using the Pearson correlation analysis. For parameters not normally distributed, statistical comparisons between groups were made using the Mann–Whitney U tests, Wilcoxon Sign Rank Test, and associations were analyzed using the Spearman correlation analysis. The categorical variables were compared using the chi-square test. Stepwise multiple regression analysis was performed to determine independent factors associated with axial length elongation of one year. *P* < 0.05 was considered statistically significant.

## Results

### Patient and treatment characteristics

Seventy-three patients and 73 eyes were included in this study. There were 26 (35.6%) males and 47(64.4%) females. The average age at the initiation of OK lens wear was 10.23 ± 1.74 (8 ~ 15) years old. The average baseline SE refractive error was −3.22 ± 1.19 (−1.00 ~ −5.75) Diopters (D), and astigmatism was less than 1.50D. Parental myopia were divided into three grades according to the degree of myopia. Grade 1: none of the parents had myopia, 48 (65.8%) cases; grade 2: one or both parents had mild to moderate myopia (≥ − 6.00D), 10 (13.7%) cases; grade 3: one or both parents had high myopia (<−6.00D), 15 (20.5%) cases.

The subjects’ baseline corneal power, CCT, ACD, AL, and corneal power, CCT, ACD, AL after lens wear for one year are listed in Table 2. Corneal power, CCT, and ACD changed by an average of-1.99 ± 0.89D (−4.00 ~ 0D), −5.95 ± 14.23 μm (−36.00 ~ 28.00 μm), and-0.034 ± 0.075 mm (−0.29 ~ 0.20 mm), respectively. AL increased by an average of 0.18 ± 0.17 mm (−0.13 ~ 0.65 mm). All the differences reached statistical significances.

**Table 2:** Comparisons of data between baseline and after one-year lens wear

	Baseline	After one year	***P***
Corneal power (D)	43.08 ± 1.15	41.09 ± 1.47	<0.001*
CCT (μm)	534.38 ± 30.97	528.44 ± 30.42	0.001*
ACD (mm)	3.70 ± 0.20	3.67 ± 0.20	<0.001*
AL (mm)	24.86 ± 0.67	25.05 ± 0.67	<0.001†

*D* Diopter, *CCT* Central corneal thickness, *ACD* Anterior chamber depth, *AL* Axial length

* Wilcoxon Sign Rank Test

†paired *t*-test.

### Correlations of axial length elongation and baseline factors

**Fig. 1:**
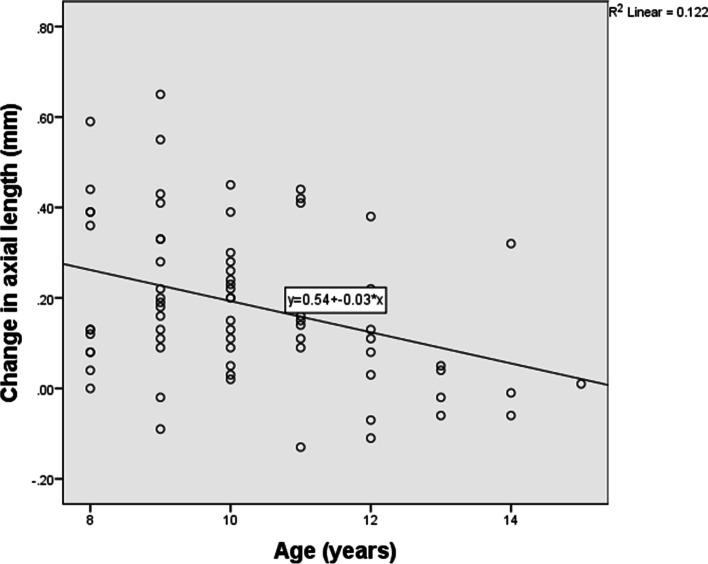
Initial age of OK lens wear had negative correlation with axial length elongation of one year

**Fig. 2:**
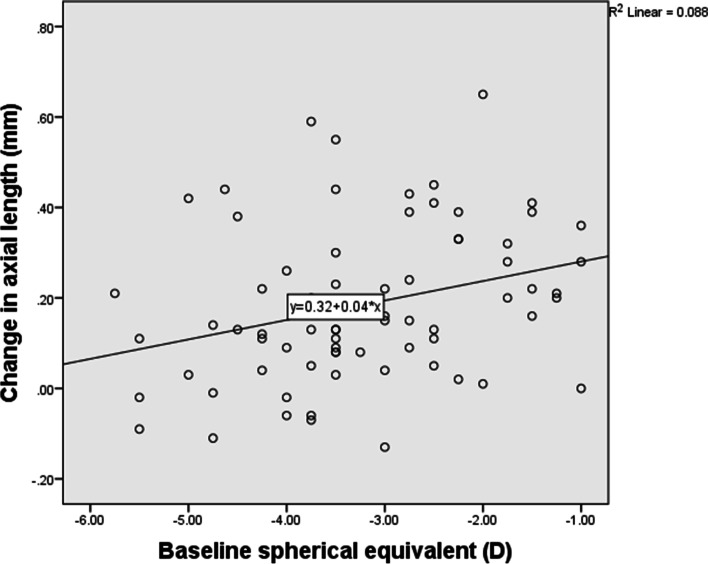
Baseline spherical equivalence of refractive error had positive correlation with axial length elongation of one year

**Fig. 3:**
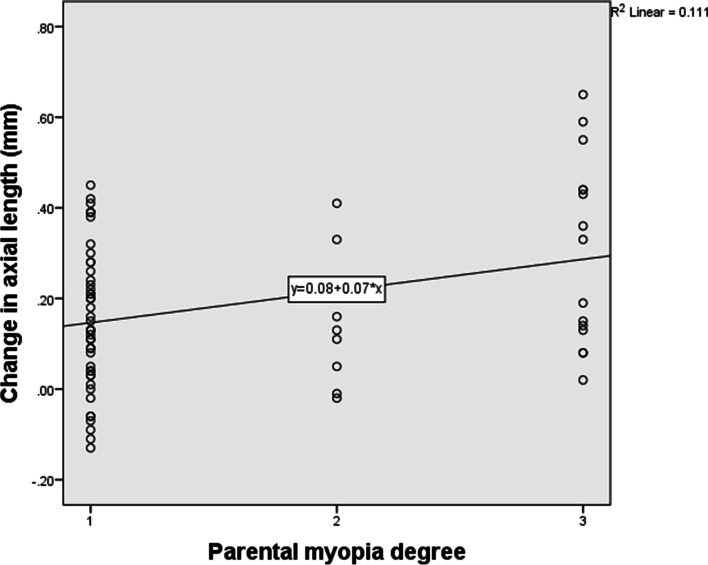
Parental myopia degree had positive correlation with axial length elongation of one year

Axial length increased by an average of 0.18 ± 0.17 mm after lens wear of one year. Correlation analysis (Table 3) showed that initial age of lens wear had significant negative correlation with AL elongation of one year. The younger the baseline age, the faster the axial elongation (Fig. 1). Baseline SE of refractive error and parental myopia degree had significant positive correlation with AL elongation of one year. Therefore, those whose myopia were lower and parental myopia were higher experienced a faster increase in AL (Fig. 2 and Fig. 3).

**Table 3:** Correlations between axial length elongation and other parameters

		*r*	*P*
Gender	26 M/47F	0.105	0.379*
Age of initial lens wear (years)	10.23 ± 1.74	−0.308	0.008*
Parental myopia degree (1/2/3)	48/10/15	0.251	0.032*
Baseline SE (D)	−3.22 ± 1.19	0.329	0.005*
Baseline corneal power (D)	43.08 ± 1.15	−0.132	0.267*
Baseline CCT (μm)	534.38 ± 30.97	0.017	0.885*
Baseline ACD (mm)	3.70 ± 0.20	−0.041	0.730*
Baseline AL (mm)	24.86 ± 0.67	−0.127	0.283†

*SE* Spherical equivalent, *D* Diopter, *CCT* Central corneal thickness, *ACD* Anterior chamber depth, *AL* Axial length, *M* Male, *F* Female

*Spearman correlation analysis

† Pearson correlation analysis.

### Stepwise multiple regression analysis

Stepwise multiple regression analysis showed that the factors associated significant with smaller increases in AL were older initial age of OK lens wear, lower degree of parental myopia and higher baseline SE of myopic refractive error. The statistical analyses are listed in Table 4.

**Table 4:** Stepwise multiple regression analysis of axial length elongation of one year and other factors

	B	95%CI	*P*
Baseline SE	0.037	0.007 ~ 0.068	0.018
Parental myopia degree	0.053	0.007 ~ 0.099	0.026
Baseline age	−0.023	−0.045 ~ −0.001	0.039

SE Spherical equivalent

### Analysis on cases of axial length reduction

Axial length decreased in nine eyes after one year of OK lens wear. There was no statistical difference between the AL of decreased and increased cases at baseline (24.86 ± 0.66 mm vs 24.86 ± 0.68 mm, One-way ANOVA, *P* = 0.995). After one year of OK lens wear, AL of the two groups were 24.80 ± 0.64 mm and 25.08 ± 0.68 mm respectively. Both the changes of AL (−0.06 ± 0.04 mmand0.22 ± 0.15 mm respectively) were statistically significant (paired *t* test, *t* = −4.542, *P* = 0.002 and paired *t* test, *t* = 11.469, *P* < 0.001 respectively). The difference of AL changes of the two groups was statistically significant (Mann-Whitney U test, *P* < 0.001). The contrasts of AL increased and decreased cases are listed in Table 5. Cases of decreased AL had significantly older initial age of lens wear, higher baseline myopia and lager baseline corneal power, when compared with cases of increased AL.

**Table 5:** Comparisons between cases of different changes of axial length

	Decreased AL	Increased AL	***P***
Gender	2 M/7F	24 M/40F	0.335*
Parental myopia(1/2/3)	7/2/0	41/8/15	0.099*
Baseline age (years)	11.89 ± 1.90	10.00 ± 1.60	0.007†
Baseline SE (D)	−4.33 ± 0.85	−3.06 ± 1.15	0.002†
Baseline corneal power (D)	43.86 ± 1.19	42.96 ± 1.11	0.028‡
Baseline CCT (μm)	520.78 ± 30.94	536.30 ± 30.73	0.161‡
Baseline ACD (mm)	3.76 ± 0.19	3.69 ± 0.19	0.364†
Baseline AL (mm)	24.86 ± 0.66	24.86 ± 0.68	0.995‡
Change of AL (mm)	−0.06 ± 0.04	0.22 ± 0.15	<0.001†

*SE* Spherical equivalent, *D* Diopter, *CCT* Central corneal thickness, *ACD* Anterior chamber depth, *AL* Axial length

* Chi-square test

†Mann-Whitney U test

‡One-way ANOVA

## Discussion

### Axial length elongation after OK lens wear

Cho et al. [6] reported in 2005 that AL elongation of myopia could be controlled by orthokeratology. At the end of the two-year study, axial elongation of OK lens group reduced by nearly 50% compared with that of single-vision glasses group. The similar results have been reported by numerous studies [7–9]. The treatment outcome was also remarkable even in high myopia children with partial reduction OK lens [10]. In former researches, the changes of AL after OK lens wear were 0.16 ± 0.17 mm [11] and 0.22 ± 0.15 mm [17] for one year, 0.29 ± 0.27 mm [6], 0.31 ± 0.27 mm [12], 0.36 ± 0.24 mm [13], 0.37 ± 0.27 mm [18] and 0.39 ± 0.27 mm [8] for two years, and 0.99 ± 0.47 mm [7] for five years. In this study, axial elongation was 0.18 ± 0.17 mm one year after OK lens wear, which was consistent with the result of previous studies. In contrast, the treatment effects were more remarkable in some researches with AL changes of one year were 0.07 ± 0.21 mm [14] and 0.08 ± 0.15 mm [15].

These two researches were contralateral comparison studies in which myopic anisometropes were recruited. The monocular myopia or the more myopic eyes were fitted with OK lenses only. The changes in AL of the contralateral eyes after one year were 0.36 ± 0.23 [14] mm and 0.39 ± 0.32 mm [15] respectively. This means that the reduction rates of AL elongation by OK lens reached to 79 and 81%, while in other studies, the reduction rates were 30% ~ 63% [6–8, 10–13]. The significant therapeutic effect may be attributed to not only the change of peripheral retinal defocusing pattern of the treated eye but also the change of relative relationship between the peripheral retinal defocusing patterns of the two eyes. Further exploration is needed to find and confirm the reasons.

### Factors relevant to AL elongation after OK lens wear

Axial length elongation after OK lens wear was reported to be significantly correlated with baseline age [11–13, 17–20], initial refractive error [19, 21], CCT and post treatment relative peripheral refractive power [17], summed corneal power change from the central to the mid-peripheral cornea [13], the magnitude of corneal relative peripheral power change [22], and pupil diameter [23]. There were also contradictory findings. Some reported that there was no significant correlation between the change of AL and initial age of OK lens wear [21, 22], nor the baseline refractive error [11–13, 18, 22]. In this study, we found that there were significant correlations between axial elongation and initial age of OK lens wear and baseline myopic spherical equivalent. Sampling error was the possible reason for different results. Nevertheless, some characteristics of OK lens treatment could be drawn from the results of these researches.

Children whose parents had higher myopia had greater AL increase in our research. It indicated the influence of genetic factors on the progression of myopia. Santodomingo-Rubido et al. [20] found that parental refraction had no significant correlations with children’s AL change. Nevertheless, lower levels of parental myopia were associated with smaller increases in children’s AL. In our study, we divided parental myopia into grades according to the degree of parental myopia. In the study of Santodomingo-Rubido et al. [20], the analysis was based on the raw data. Although the results of significance test were different, the trends of correlation were similar.

The law of myopia development after OK lens wear is in consistent with that of natural progression of myopia in children. Myopia development and axial elongation were faster in younger children [24–26] and those who had myopic parents [25, 27]. However, the children with higher base myopia had faster myopia progression [24], which was in contrast with that after OK lenses wear [19, 21]. The causation may be that to treat higher myopia the central corneal power should be reduced more, thus mid-peripheral corneal power will increase more and may induce more peripheral retina myopia defocus. Moreover, is it possible to get steeper mid-peripheral corneal power through lens design, so as to obtain more significant therapeutic effect? A short-term study of different compression factors found that no significant changes in eye parameters were found between the different design lenses [28]. However, OK lens of smaller back optic zone diameter achieved better curative effect [29, 30]. Further investigations will be need to make OK lens more effective in myopia control.

Since the younger children with myopic parents are prone to have axial elongation after OK lens treatment, more aggressive or combined treatment should be considered. For example, combined orthokeratology and 0.01% atropine solution could achieve better therapeutic effect [31, 32], especially in children with low initial myopia [32].

### Possible causes of negative axial length elongation

Axial length reduction after OK lens wear is an interesting phenomenon. It is common but rarely noticed. In some studies, overall AL increased but some cases decreased [9–15, 17–19, 21, 22, 33]. Moreover, in some studies, even the overall means decreased [16, 34]. Again, a few studies have investigated on the AL shortening issue. We made some comparative studies on the cases of positive and negative axial growth. We found that compared with patients with increased AL, patients with decreased AL had significantly older age, higher baseline myopia and higher baseline corneal power. That was in correspondence with the law who were less axial elongation after OK lens wear. In the studies that found AL decreased, the subjects were older children (10.8 ~ 17.0 years) [16] or young adults (25.62 ± 3.57 years) [34] whose progression of AL were slow or stopped. The AL shortening effect of the OK lens would not be concealed by the natural growth of the eyeball. Moreover, it was more obvious in young adults whose eyeball growth stopped [34].

The possible reasons of negative growth of AL after OK lens wear have not been addressed in most previous studies. They were only discussed in some researches and often thought to be an illusion of thinning of central cornea and thickening of choroid [9, 14–16, 21]. CCT thinned about 10 μm [35–38] and choroid thickened about 20 μm after OK lens wear [38–40]. We found that CCT became thinner by 5.95 ± 14.23 μm, and for the cases of AL decreased, CCT thinned 9.44 ± 13.28 μm. But AL decreased 0.06 ± 0.04 mm. Although choroidal thickness was not measured at the same time, the amount of AL reduction did not seem to match the amount of choroidal thickening and CCT thinning according to previous studies. González-Mesa et al. [34] found that AL decreased by 75 μm and 160 μm after OK lens wear for 15 days and 12 months in young adults. CCT value was subtracted from AL value to correct for the influence of CCT on AL change. If the decrease of AL was only due to the thickening of choroid, such large amount of choroidal thickening has never been reported. There were cases of AL decrease even after one month of OK lens cessation, when ocular biometric parameters all regressed to the baseline [40]. In addition, there seems to be evidence to support this in a study that axial shortening could not be fully explained by central corneal thinning and choroidal thickening [28].

Further studies are needed to confirm whether there are other causes of axial shortening other than corneal thinning and choroidal thickening. Some studies reported that ACD decreased significantly after OK lens wear [20, 28, 34]. We found that ACD decreased 0.034 ± 0.075 mm (*P* < 0.001). ACD increased with age in children who were not treated by OK lens [41]. In addition, some researchers found that posterior cornea flattened significantly [34, 42]. We hypothesize that the changes of anterior segment structure after OK lens wear may indicate the change of the eyeball shape, e.g., it may change from prolate to oblate. This causes the sagittal axis of the eyeball, which is commonly referred to as the ocular axis, to become shorter. However, this change may be masked by the fast growth of eyeball. Therefore, it appears more in children with slow axial elongation and adults whose eyeball growth has been stopped. As far as we know, this is the first paper that targeted research was done to understand the characteristics of cases with negative axial growth after OK lens wear. It mentions the possible role of OK lens in regulating the shape of eyeball. Further investigations are required to prove the hypothesis and elucidate the underlying mechanism.

### Limitation of this study

Lack of sufficient and direct evidence to support the viewpoint is the most notably deficiency of this paper. As a retrospective study, we could only analyze the existing previous data. Prospective design researches that include comprehensive data and evidence of ocular morphological changes are needed to confirm the hypothesis. The second deficiency of this paper is that there was no control group. Although it was only a correlation study, if we could do some correlation analysis in the control group, we could compare the development trend of myopia under different treatment measures. The third deficiency of this paper is that the sample size was a little small. Because several different brands of OK lenses were used at the refractive clinic at the same time, only the patients wore one brand of OK lens were selected for research.

## Conclusions

Despite its limitations, this study is the first research to investigate axial length decrease after OK lens treatment, and presents the hypothesis that there may be causes other than central corneal thinning and choroidal thickening, which need further study to confirm. Although the retardation effect of OK lens on myopia progression has been widely recognized, some myopia children still have fast axial elongation after OK lens treatment. Myopia children with lower baseline myopia, younger initial age and higher parental myopia are prone to have faster axial elongation. More aggressive or combined treatment should be considered.

## Data Availability

The datasets used and/or analysed during the current study are available from the corresponding author on reasonable request.

## Abbreviations

OK: Orthokeratology
SE: Spherical equivalent
CCT: Central corneal thickness
ACD: Depth of anterior chamber
AL: Axial length
D: Diopter
M: Male
F: Female
